# Variability and Innovation in Faculty Development Models in Emergency Medicine

**DOI:** 10.1002/aet2.70232

**Published:** 2026-08-11

**Authors:** Jessica Baez, Susan Promes, Nicolas Ellis, Kay Leaming‐Van Zandt, Joan Noelker, Robbie Paulsen, Douglas Char, Lawrence Kass, Elizabeth Leenellett, Erin McDonough, Sally A. Santen

**Affiliations:** ^1^ Department of Emergency Medicine University of Cincinnati Cincinnati USA; ^2^ Department of Emergency Medicine Penn State University University Park USA; ^3^ Department of Emergency Medicine Washington University in St. Louis Washington USA

## Abstract

**Background:**

Ongoing faculty development is essential in academic emergency medicine (EM) to ensure physicians remain clinically competent, educationally effective, and aligned with evolving healthcare demands. Despite this necessity, approaches to faculty development vary widely in structure, participation expectations, and underlying educational philosophy.

**Objectives:**

This paper explores the spectrum of faculty development models, including voluntary participation, asynchronous learning, and institutional mandates, examining how each aligns with learning theory and motivational frameworks such as Self‐Determination Theory.

**Discussion:**

We discuss the benefits and limitations of different methods, including their impact on engagement, skill retention, accountability, and institutional culture. Drawing from both educational literature and implementation experience, we highlight the importance of intentional design, learner autonomy, and system‐level alignment in promoting meaningful and sustainable faculty growth.

**Conclusion:**

Ultimately, the paper presents an expert‐informed conceptual framework to guide academic departments in selecting and tailoring faculty development strategies that balance individual motivation with organizational responsibility.

## 1 Introduction

As healthcare evolves and clinical demands grow more complex, the responsibility of academic emergency medicine (EM) faculty to maintain their clinical competency is increasingly important. In response, the Accreditation Council for Graduate Medical Education (ACGME) and the American Board of Medical Specialties (ABMS) have emphasized the importance of lifelong learning and continuous professional development to ensure safe and effective patient care [1, 2].

However, the obligation to remain competent extends beyond accreditation or institutional policy. As Santen et al. [3, 4] and Nelson et al. [5] argue, physicians carry both a moral and professional responsibility to remain current and competent, not only for the benefit of their learners but also out of duty to their patients and the public. Faculty serve not only as clinical providers but as gatekeepers of safe practice and role models for future physicians. When a physician's competency wanes, patient care may suffer. This can ultimately lead to an erosion in public trust of the medical field. Thus, for both the patients and the public, individuals may be expected to undergo remedial training secondary to a poor patient outcome [6]. Accountability, therefore, is both an individual and system responsibility that hopes to address skill decline prior to patient impact.

Yet the pathway to maintaining and improving skills is rarely straightforward. As highlighted in the literature, learning and skill acquisition in the health professions follows complex, non‐linear trajectories [7]. Individual clinicians demonstrate variable learning curves, influenced by prior experience, context, feedback, and opportunities for deliberate practice [7]. In contrast to the outdated assumption that competence, once achieved, remains static, the reality is that skills may decay over time without intentional reinforcement. Faculty development must be conceived as an ongoing, adaptive process, one that supports physician educators in maintaining performance, adapting to new clinical challenges, and continuing to grow over the span of their careers.

Pusic and colleagues also underscore the value of tracking progress over time, a principle applicable not only to learners but to faculty as well [7]. Monitoring skill performance longitudinally can help institutions identify where reinforcement or remediation is needed. Faculty development programs that include deliberate practice, simulation, and performance assessment align with these educational principles and can support sustained clinical excellence.

Despite these compelling arguments, faculty development offerings in EM remain variable in structure, implementation, and expectations. From the workshop “Developing a Clinical Skills Training Framework for Emergency Medicine Faculty” that was hosted at the Society for Academic Emergency Medicine's (SAEM) annual meeting in 2025, it was revealed that some institutions rely on voluntary participation. There is a trust in faculty members' intrinsic motivation and professional commitment, and this is supported by a survey of Academic Chairs [8]. Other institutions may mandate engagement or tie it to incentives such as Continuing Medical Education (CME) credits, promotion, or clinical privileges. A national needs assessment by Karademos et al. [9] further illustrates the heterogeneity in faculty development across academic EM, with wide variation in goals, formats, and accountability mechanisms.

This concept paper arose from collaborative development, presentation, and discussions during the SAEM 2025 Annual Meeting workshop, *Developing a Clinical Skills Training Framework for Emergency Medicine Faculty*. The purpose of this manuscript is to present an expert‐informed conceptual framework for faculty development in EM, discuss these approaches through the lens of educational theory, and highlight practical considerations for institutions seeking to strengthen faculty development efforts.

## 2 Methodology

Relevant literature was identified through a targeted, non‐systematic search of PubMed/MEDLINE and Google Scholar. Search terms included combinations of keywords such as *emergency medicine*, *faculty development*, *medical education framework*, *clinical skills training*, and *continuing professional development*. Additional sources were identified through screening of reference lists from key manuscripts and author subject‐matter expertise. Literature was selected based on relevance to faculty development program design, implementation strategies, and evaluation methods in graduate medical education. No formal inclusion or exclusion criteria were applied, and study quality appraisal was not performed, consistent with the conceptual intent of the work.

This manuscript was developed following the SAEM 2025 Annual Meeting workshop, *Developing a Clinical Skills Training Framework for Emergency Medicine Faculty*. The manuscript reflects key themes identified through workshop development, discussion among faculty development experts, and review of relevant literature. The intent was not to conduct a formal systematic or narrative review, but rather to develop an expert‐informed conceptual framework describing factors that may influence faculty development program design in emergency medicine. Both the manuscript and didactic were developed through a collaborative, iterative approach by a working group of academic emergency physicians with interest and experience in faculty development. Contributors were identified based on their roles in faculty education, simulation, and academic leadership.

## 3 Conceptual Framework for Faculty Development

Based on workshop discussions of faculty development experts, review of existing educational theory and the literature including faculty development, and the authors' collective experience in faculty development, a set of interrelated domains emerged as central to faculty development program design. These include educational goals and intended outcomes, institutional context and available resources, participation models, educational delivery methods, program evaluation, and ongoing refinement [6, 7, 9]. Together, these elements form a conceptual framework intended to support departments in aligning faculty development efforts with local needs, priorities, and constraints.

Educational goals and intended outcomes represent the foundational driver of faculty development design and should be defined early in the planning process. These goals shape decisions regarding participation models, educational delivery methods, and evaluation strategies. Common goals include maintenance of procedural competency, teaching skill development, onboarding of new faculty, remediation, credentialing and certification requirements, leadership development, and scholarly growth.

Program evaluation should be explicitly aligned with these intended outcomes [10]. Potential measures include participation rates, satisfaction, perceived competence, behavioral change, procedural performance, and attainment of program‐specific objectives [10, 11, 12]. Consistent with Self‐Determination Theory, institutions may also consider outcomes such as faculty engagement and willingness to participate in future development activities [12].

**FIGURE 1:**
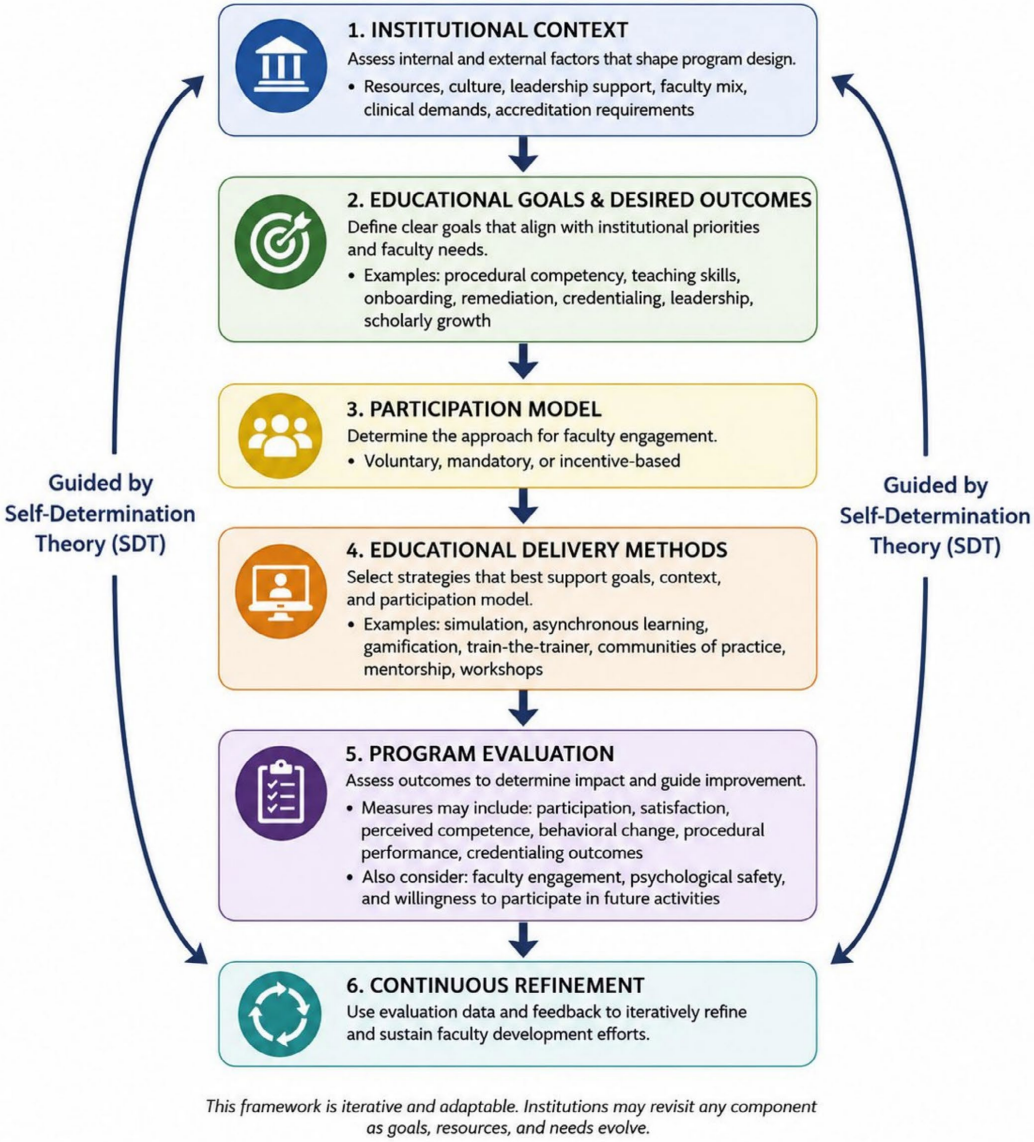
Implementation framework for faculty development program design in emergency medicine.

The remaining components of the framework which include institutional context, participation models, educational delivery methods, and iterative refinement are interdependent and should be selected in alignment with clearly defined educational goals and evaluation strategies. The following sections describe each component and their relationships in greater detail (Figure 1).

The proposed framework aligns with established implementation and evaluation models that may guide its application in practice. From an implementation perspective, the framework is consistent with the Consolidated Framework for Implementation Research (CFIR) [13] and the RE‐AIM framework [14] in considering contextual determinants and implementation outcomes such as reach, adoption, and sustainability. From an evaluation standpoint, outcomes can be broadly mapped to Kirkpatrick's levels of evaluation [10] and Moore's expanded outcomes framework [11], ranging from learner reaction and knowledge acquisition to behavior change and programmatic impact. Consistent with Self‐Determination Theory, evaluation may also include learner‐centered outcomes such as engagement, psychological safety, perceived autonomy, buy‐in, and likelihood of continued participation [12].

## 4 Models of Participation of Faculty in Development Programs

Faculty development programs in academic emergency medicine vary widely in structure and expectations, reflecting differing philosophies about how best to support professional growth. Participation models can be broadly categorized as voluntary, mandatory, or incentive‐based—each with unique advantages, limitations, and implications for faculty engagement. Understanding these models through the lens of motivation theory, institutional accountability, and educational outcomes allows for a more nuanced approach to program design. The following sections explore how each model shapes faculty participation, skill maintenance, and institutional culture.

### 4.1 Voluntary Participation and Intrinsic Motivation

Voluntary faculty development models are often predicated on the assumption that physicians, as highly educated professionals, possess an inherent drive toward continuous improvement and self‐regulation. This perspective aligns closely with Self‐Determination Theory (SDT), a psychological framework developed by Ryan and Deci, which posits that intrinsic motivation, the internal drive to engage in an activity for its inherent satisfaction, is nurtured when three basic psychological needs are met: autonomy, competence, and relatedness [12].

Voluntary models support autonomy by allowing faculty to choose when, how, and what they learn, aligning development activities with individual interests, learning preferences, and schedules. This sense of volitional control has been consistently linked to higher engagement, persistence, and satisfaction in educational settings [12]. Moreover, when faculty identify personal knowledge or skill gaps and proactively pursue resources to address them, they reinforce their sense of competence, a key driver of intrinsic motivation and professional identity.

The third SDT component, relatedness, is also supported in well‐designed voluntary systems that create opportunities for collaboration and peer learning. When development activities are embedded within a supportive and collegial environment, faculty are more likely to experience a sense of connection with peers, reinforcing shared goals of clinical excellence and teaching effectiveness. In this way, voluntary models can foster a culture of growth that is both individualized and socially cohesive [15, 16].

However, relying solely on intrinsic motivation can result in inconsistent participation and skill degradation, particularly for procedural competencies that require regular reinforcement. Faculty who are less self‐directed, less engaged, or unaware of their own developmental needs may be overlooked in voluntary systems [17]. Kohan et al. suggest that while voluntary individualized approaches can be effective, they require intentional design and alignment with institutional goals to avoid fragmentation and ensure educational impact [18]. Without structured expectations or accountability, voluntary models risk excluding those who may benefit most from development opportunities. Studies in learning science emphasize that without deliberate practice and feedback, both knowledge and skill are susceptible to decay over time [7].

Thus, while SDT provides a compelling rationale for leveraging intrinsic motivation through voluntary development, it also underscores the need for balance. Voluntary models should be designed not just to promote autonomy, but to intentionally support competence and relatedness, and to safeguard educational quality through institutional scaffolding, clear expectations, and access to feedback‐rich learning experiences.

### 4.2 Mandatory Models and Procedural Accountability

Mandatory participation in faculty development, sometimes reinforced by incentives or by disincentives for non‐participation, aims to ensure safe supervision and promote standardized clinical practice, particularly for procedural competency. These models are typically reinforced by external motivators such as institutional policies, credentialing requirements, and alignment with promotional criteria. Additional incentives like Continuing Medical Education (CME) credits or value‐based monetary compensation can further encourage participation (see below). As noted by Clyne et al., structured validation processes, such as requiring simulation‐based assessments prior to supervising high‐stakes procedures, are becoming increasingly common [19]. These systems acknowledge that competency is not static and recognize that procedural skill attrition can occur without periodic reinforcement. External motivators can thus play a critical role in maintaining baseline competence and engaging faculty who might not otherwise prioritize development activities.

Mandatory participation may also be driven by institutional risk management or by credentialing requirements from hospital committees or certifying bodies. While these systems enhance accountability and reflect broader demands for public trust, patient safety, and institutional oversight, they can provoke resistance if perceived as punitive, overly bureaucratic, or misaligned with faculty values. However, when implemented transparently and supported by leadership, mandatory models can be particularly effective when they align with faculty goals and professional identity [20]. As studies have emphasized, skill acquisition and maintenance are non‐linear and context‐sensitive; rigid structures may fail to accommodate individual variation or professional autonomy [7, 21]. Therefore, mandatory models are most effective when paired with feedback‐rich environments and opportunities for individualized learning, balancing enforcement with educational support [18].

### 4.3 Incentive‐Based Models

Incentive‐based models offer an alternative approach to fostering faculty engagement by leveraging external motivators to encourage participation in faculty development activities, particularly those focused on teaching effectiveness, clinical competency, and procedural skill maintenance. These models use tools such as financial compensation, allocated time, continuous medical education (CME) or maintenance of certification (MOC) credits, promotion eligibility, and institutional recognition to incentivize engagement. In clinical environments where patient care demands compete with academic responsibilities, well‐designed incentives can help elevate the priority of faculty development activities [22]. A 2006 systematic review of academic incentive compensation programs found associations with increased productivity and academic output [23], while more recent studies confirm that even modest rewards can improve faculty participation in teaching and procedural training efforts [24, 25]. The American Board of Medical Specialties (ABMS) continuing certification standards exemplify an incentive‐driven, mandatory framework that blends positive and negative reinforcement: active participation can maintain one's certification status, while non‐compliance may result in credentialing consequences [2]. Likewise, some institutions tie procedural privileges or supervisory roles to the completion of faculty development requirements, further reinforcing participation as a component of clinical accountability and institutional alignment.

Although incentives can effectively promote initial participation, they do not inherently foster sustained engagement or long‐term behavioral change [26]. According to self‐determination theory, extrinsic rewards may diminish internal motivation if perceived as coercive or misaligned with individual or professional values [27, 28]. Incentive‐based strategies are most effective when they augment, rather than supplant, intrinsic motivators such as autonomy, purpose, and mastery [12]. To mitigate this risk, incentive models should be designed to offer rewards that are perceived as meaningful, equitable, and consistent with institutional and professional norms. Otherwise, they risk being seen as transactional rather than transformational, potentially undermining long‐term professional growth. Incorporating flexibility and personalization, such as linking development activities to promotion, offering a selection of reward options (e.g., stipends, recognition, professional development opportunities), or bundling participation with MOC or CME credits, can reduce barriers to engagement while supporting institutional goals in safety, quality improvement, and academic excellence.

### 4.4 Summary of Models

Taken together, voluntary, mandatory, and incentive‐based models each play a distinct yet complementary role in advancing faculty development and ensuring clinical competency. Voluntary participation supports autonomy and appeals to intrinsically motivated faculty but may lead to variable uptake. Mandatory models ensure standardization and accountability, especially for high‐stakes procedural skills, but risk disengagement if overly rigid. Incentive‐based approaches bridge these models by motivating participation through structured yet flexible rewards. When thoughtfully integrated, these frameworks can collectively foster a culture of continuous learning, shared responsibility, and sustained professional growth.

## 5 Methods of Providing Faculty Development

The delivery of faculty development in emergency medicine requires thoughtful alignment between educational goals, instructional strategies, and the unique constraints of clinical practice. As the demands on faculty continue to grow, programs must offer flexible, evidence‐based methods that not only reinforce clinical competence but also foster teaching effectiveness, empathy, and professional growth. This section explores a range of faculty development approaches including simulation and deliberate practice, informal practice opportunities, gamification, and train‐the‐trainer models. This highlights both established and emerging strategies to enhance learning, engagement, and sustainability across diverse settings.

### 5.1 Simulation and Deliberate Practice

Simulation‐based training, when paired with deliberate practice, is one of the most evidence‐supported methods for procedural skill maintenance. Okuda et al. and McGaghie et al. highlight the value of feedback, repetition, and psychological safety in these learning environments [29, 30].

Given the widespread integration of simulation in residency training, most faculty are already familiar with it, easing potential barriers to buy‐in [31]. Faculty are also keen on incorporating simulation into their development programs, with evidence indicating that it enhances procedural skills which are prone to attrition over time [19, 32]. This attrition is particularly notable in pediatric skills and certain rare procedures [32, 33]. Studies demonstrate the efficacy of simulation for various critical tasks, including high acuity, low occurrence (HALO) procedures, critical care, difficult conversations, and teamwork [34, 35, 36, 37, 38, 39]. Further research highlights improved performance in CVC placement, which not only boosts competence but also improves patient safety through the reduction of risks like CLABSI [34, 36].

Faculty development programs also benefit from simulation and can help to improve empathetic care and reduce gender bias [40]. Peer coaching for debriefs has been shown to be particularly effective, and faculty tend to prefer working with peers rather than trainees [11, 41] when honing their own skills. Suggested approaches include faculty bootcamps for procedure teaching in pediatric emergency medicine [42]. Simulation not only enhances procedural skills but also can improve both attitudes and behaviors of faculty members [41, 43]. Faculty exhibit better performance in regional blocks, procedural teaching, and transvenous pacing after simulation‐based training [44, 45].

Innovative modalities such as virtual reality (VR), in situ simulation, and artificial intelligence (AI)–based training provide effective avenues for faculty development [46, 47, 48, 49, 50]. Approaches like virtual debriefs and self‐directed simulation show promise, though teledebriefing appears less favorable [50, 51]. In situ simulation, in particular, has demonstrated both effectiveness and feasibility [52, 53]. Nontraditional strategies, including AI‐driven simulation for suicide risk assessment and 3D printing for cost‐effective training models, can help overcome common barriers such as time and space constraints, cost, and the need for instructor training [5, 34, 54, 55, 56, 57, 58]. Establishing dedicated simulation programs further addresses these challenges and supports consistent, high‐quality faculty development [41].

Content delivery may come in the form of “open office hours” or procedural practice labs allowing for just‐in‐time refreshers. However, as Bell et al. found, expectations for procedural competency are often poorly defined, and practice opportunities may be inconsistently utilized [21]. Providing opportunities to practice critical skills is only part of the solution. What and how faculty “practice” HALO and more routine skills (central lines, intubation) are critical to the faculty member feeling successful and prepared. Using checklists, making use of video recordings of intubations that can be played back and critiqued, testing knowledge of complex skill performance are all necessary. Procedure updates and practice sessions allow faculty to adopt new approaches and update their knowledge based on current practice in a low‐stakes, supportive environment.

It is often overlooked but faculty must not only be prepared to perform the specific skill, but they must be able to teach it as well. Over time their ability to provide clear, actionable feedback to trainees becomes more challenging. Variability in practice is often encouraged but especially for novice learners, the use of consistent, explicit, shared messaging by multiple faculty is critical as they begin to master the procedure. This is why near‐peer feedback is often what is most desired by trainees. The longer it has been since physicians performed a complex task, the more difficult it may be to identify subtle procedural steps and provide specific feedback to the bedside learner [7].

### 5.2 Asynchronous Learning

Asynchronous learning opportunities have emerged as a flexible and scalable method of faculty development, offering a wide range of formats including web‐based modules, recorded lectures, procedural videos, podcasts, and blogs that can be accessed on demand. These formats are particularly appealing to busy academic emergency physicians, who must balance clinical duties, teaching, research, and personal obligations. By allowing learners to engage with material at their own pace and on their own schedule, asynchronous modalities uphold the autonomy central to SDT while also offering opportunities to enhance competence through repetition, feedback, and targeted content review [12].

One evidence‐supported asynchronous strategy is mental practice; a cognitive rehearsal technique used widely in sports and now gaining traction in medicine. Mental practice involves the internal visualization of procedural or clinical steps and has been shown to improve performance, reduce anxiety, and reinforce skill retention in emergency medicine [59]. When paired with video instruction or simulation‐based content, asynchronous mental practice offers a low‐cost, high‐yield method for procedural skill maintenance, particularly for rare or high‐risk interventions.

E‐learning modules are another cornerstone of asynchronous faculty development. These may be institutionally mandated content areas such as sedation safety or blood product administration, and they may be paired with post‐module assessments or examinations to ensure faculty meet certain competency standards. While initially designed for compliance, these modules can serve a deeper educational purpose when crafted with deliberate learning objectives and opportunities for self‐assessment. Studies have shown that internet‐based e‐learning can improve clinician behavior and patient outcomes when implemented effectively, particularly when it includes interactive and learner‐centered design elements [60].

In addition to institutional content, many faculty members turn to on‐demand procedural videos to refresh their memory before performing infrequent or high‐risk skills. These resources offer concise, focused reviews and can be easily integrated into just‐in‐time learning strategies. Furthermore, faculty increasingly engage with podcasts and blogs, which offer convenient access to cutting‐edge medical education, emerging clinical practices, and expert opinion. Asynchronous media such as these may be effective and can be preferred by many faculty members. A national study found that emergency medicine faculty strongly favored flexible, self‐directed educational formats that accommodated variable schedules and learning styles [61].

The timing of content delivery also plays a role in knowledge acquisition because learners retain more knowledge when podcast content is spaced over time rather than delivered all at once, a concept aligned with the spacing effect in cognitive psychology [62, 63]. These findings support the idea that asynchronous faculty development can be optimized not just through format variety, but also through intentional sequencing and reinforcement strategies.

Despite their advantages, asynchronous models also require thoughtful design to ensure they are not passive or perfunctory. Without opportunities for feedback, practice, or application, faculty may complete modules without meaningful learning. Therefore, integrating asynchronous methods with deliberate practice principles, competency assessments, and peer engagement can help bridge this gap.

Overall, asynchronous faculty development methods offer a powerful means of supporting lifelong learning. When guided by the principles of SDT, promoting autonomy, reinforcing competence through personalized repetition and feedback, and fostering relatedness via shared learning resources, they can be both effective and sustainable. Institutions that invest in well‐designed asynchronous platforms, including mental practice tools, procedural video libraries, and interactive modules, can provide faculty with accessible, relevant, and enduring opportunities for clinical growth.

### 5.3 Gamification and Innovative Approaches

Gamification, the application of game‐design elements in non‐game contexts, has emerged as a compelling strategy to improve motivation and engagement in faculty development programs. By integrating features such as point systems, leaderboards, team‐based challenges, and achievement badges, gamification can create interactive learning environments that foster intrinsic motivation and enhance knowledge retention.

Gamification is especially well‐suited to the emergency medicine context, where high‐paced, problem‐solving environments align with the motivational elements of game‐based learning. Although most published gamified faculty development initiatives exist outside of EM faculty development, they demonstrate promising results. For instance, applying gamified methods in health professions education has been shown to improve learner participation, motivation, and performance, with additional benefits including increased collaboration and social engagement [64, 65].

In a review of faculty development in higher education, Noe et al. emphasize that gamification supports active learning by encouraging iterative engagement and immediate feedback loops [66]. These elements echo principles from adult learning theory, particularly self‐directedness and experiential learning, making gamification a natural fit for time‐constrained faculty who benefit from modular, interactive learning tools.

In healthcare settings, gamification has also been used to encourage behavioral change and professional growth among providers. For example, game‐based CME programs and team competitions have increased participation in quality improvement initiatives and guideline adherence [64]. Gamified formats may help reduce psychological resistance to training mandates, as learners perceive them as enjoyable and less hierarchical. However, to be effective, gamified approaches must be thoughtfully designed. Poor implementation can trivialize content or create disparities in access or engagement. Best practices include aligning game mechanics with specific educational objectives, fostering collaboration rather than pure competition, and embedding reflection and debriefing elements to consolidate learning [64, 65, 66].

While empirical research specific to gamified faculty development in emergency medicine is limited, the broader literature supports its utility in faculty training across disciplines. Given EM's fast‐paced culture and familiarity with simulation, the specialty is uniquely positioned to innovate with gamification as a faculty development tool.

### 5.4 Train‐The‐Trainer Models

To scale faculty development, some departments adopt train‐the‐trainer approaches. Lavoie et al. describe a pediatric EM bootcamp model that prepares faculty to teach and supervise more effectively, reducing reliance on a small group of educational leaders [42]. Butki et al. describe a multi‐site train‐the‐trainer pilot program to teach point‐of‐care ultrasound (POCUS) in EM where a POCUS expert trains novice faculty in POCUS who in turn teach EM residents, resulting in very high satisfaction ratings for both trainers and learners [67]. Servey et al. take this concept to another level in describing a process by which a group of faculty across 15 specialties receive intensive facilitator training and then disseminate their teaching expertise across the institution, allowing an exponentially expanded and highly rated faculty development program [68].

The train‐the‐trainer model is also highly effective in building a local pool of trained instructors in a resource‐limited setting, as Feltes et al. describe in building a sustainable ACLS course in Rwanda [69]. While widely used in established programs such as ACLS, train‐the‐trainer approaches can be labor‐intensive to launch, may suffer from variability in trainer quality over time, and often face challenges in keeping instructors incentivized and engaged. Despite these limitations, the above examples demonstrate that train‐the‐trainer models in medical education and faculty development expand the pool of local experts available to disseminate skills to others, allowing for sustainable and more broadly reaching educational programs.

## 6 Strategic Approaches and Future Directions in Faculty Development

### 6.1 Proactive and Reactive Needs Assessment

While many faculty development efforts are reactive, responding to adverse outcomes or learner complaints, proactive needs assessments can help anticipate gaps. Farley et al. offer an example of how proactive surveys can not only identify priority content areas for faculty development but can also expose mismatches between faculty needs and existing resources [70]. Studies by Karademos and Clyne highlight the importance of data‐informed design [9, 19]. Baez et al. combine proactive and reactive approaches in proactively soliciting faculty feedback via a needs assessment survey while also reactively incorporating feedback from residents, faculty, and consulting services, often via the quality improvement and peer review process [71]. Proactive and reactive needs assessments, often in combination, ultimately provide more appropriately targeted and relevant faculty development programs addressing both real and perceived needs in faculty education.

### 6.2 Broader Educational Development: Teaching Skills, Scholarship, and Feedback

While procedural and clinical skills are essential, faculty development in EM must also address the broader competencies required of effective educators. These include instructional design, giving feedback, bedside teaching, educational scholarship, and understanding emerging trends in medical education. This is by no means unique to EM, and models for interdisciplinary faculty development and evaluation have been well described [72, 73].

Faculty are increasingly expected to be not only content experts but also skilled facilitators of learning. Workshops on topics such as adult learning theory, curriculum development, and learner assessment can help faculty refine their teaching strategies. Institutions that offer longitudinal educator development tracks or medical education fellowships provide structured pathways for faculty interested in advancing their teaching careers.

Feedback delivery is a critical area of focus. Effective feedback promotes learner growth, but many faculty report discomfort or lack of training in this area. Targeted sessions on feedback models, such as the “ask‐tell‐ask,” “advocacy‐inquiry,” or “reflective feedback conversation,” can improve both the confidence and effectiveness of clinical educators [74, 75].

Staying current with educational research is another core component of faculty development. Journal clubs, education grand rounds, and digital learning communities can expose faculty to the latest evidence on topics such as competency‐based medical education, learning analytics, and assessment strategies. These efforts also support scholarly productivity, helping faculty contribute to the growing body of EM education literature.

Finally, cultivating a community of practice among faculty educators can promote shared learning and mentorship. Departments that foster collaboration, provide recognition for educational work, and offer opportunities for faculty to lead educational innovation often see higher engagement and impact in their development programs.

### 6.3 Implications for Education and Training in Emergency Medicine

The current landscape of faculty development in EM is highly variable, influenced by institutional culture, resource availability, and regulatory requirements. As a result of this, those interested in implementing faculty development efforts must take care to evaluate the current state of faculty development in their institution, identify unmet needs, and consider which approach is likely to be the best fit for their circumstances.

While voluntary models may foster autonomy and engagement, they risk inconsistency. Mandatory or incentivized models improve participation but may generate resistance if perceived as top‐down mandates. Simulation‐based training and train‐the‐trainer programs show promise in sustaining competency and distributing faculty development responsibilities more equitably but can be resource‐intensive or require extensive faculty time.

While this framework was developed primarily within an academic emergency department context, many of its core components are intended to be adaptable to non‐academic and community settings. We recognize that community emergency departments often have different staffing models, fewer embedded educational resources, and variable access to simulation, faculty development infrastructure, and protected teaching time. As such, implementation in these environments may require simplification of participation structures, greater reliance on regional or system‐level faculty development resources, and integration into existing operational workflows rather than standalone educational programs. Conversely, certain elements of the framework like those related to defining educational goals, aligning interventions with measurable outcomes, and selecting feasible evaluation strategies are intentionally designed to be scalable across settings. Future work should specifically evaluate implementation in community and rural emergency departments to better define necessary adaptations and identify context‐specific facilitators and barriers.

For those institutions with existing faculty development initiatives, ongoing evaluation of the program post‐implementation is key to ensure it continues to meet faculty and institutional needs. Faculty development must be adaptive, responding to changing clinical environments, evolving educational standards, and the needs of both learners and faculty. Institutions should consider blending multiple models to achieve a balance between flexibility, accountability, and educational impact. The framework presented in this manuscript is intended to provide a conceptual approach to faculty development program design rather than a prescriptive implementation guide. Although informed by educational theory, workshop discussions, author expertise, and relevant literature, it has not been formally validated. Future work may explore application of implementation science frameworks, development of operational decision‐support tools, evaluation strategies, and adaptation of the framework across diverse practice environments, including community and resource‐limited settings.

Faculty development in emergency medicine is essential for ensuring that clinical educators maintain both their teaching skills and procedural competency. Institutions employ a range of strategies—voluntary, required, incentivized, and simulation‐based—to meet this need. Given the variability in models and the evolving expectations of educators and certifying bodies, further research is needed to identify best practices and optimize faculty development outcomes across the specialty. Ultimately, investing in faculty development is not only an educational imperative but a patient safety initiative (Table 1).

**TABLE 1:** Summary of most common faculty development methods.

Method of delivery	Summary	Benefits	Challenges
Simulation and Deliberate Practice	Hands‐on training using simulated scenarios and focused repetition. May be structured or low‐stakes for skill refreshers and peer feedback.	Enhances procedural skills and supports autonomy; supported by a strong evidence base; facilitates adoption of evolving clinical practices	Requires access to equipment and space; time‐intensive for faculty facilitators, relies on intrinsic motivation unless incentivized
Asynchronous Learning	Self‐paced learning via videos, podcasts, blogs, mental practice guides, and required modules or exams	Supports autonomy and standardization	Limited feedback and engagement; requires access to and development of the asynchronous content
Gamification and Innovative Approaches	Use of game elements or novel tools to create interactive learning experiences.	Increases engagement; often perceived as enjoyable and motivating	May reduce educational value if poorly designed or misaligned with learning goals; not ideal for all types of learning
Train‐the‐trainer Models	Faculty are trained to teach peers, scaling education through internal expertise.	Distributes teaching responsibilities; promotes peer learning	Requires upfront investment in training faculty champions, introduces variability in educational administration

## Author Contributions

**Jessica Baez:** conceptualization, investigation, writing – original draft, methodology, writing – review and editing, visualization, project administration, data curation, supervision, resources. **Robbie Paulsen:** writing – original draft, writing – review and editing. **Sally A. Santen:** conceptualization, writing – original draft, methodology, visualization, writing – review and editing, supervision. **Susan Promes:** conceptualization, writing – original draft, writing – review and editing. **Nicolas Ellis:** writing – original draft, writing – review and editing. **Elizabeth Leenellett:** writing – original draft, writing – review and editing. **Douglas Char:** writing – original draft, writing – review and editing. **Lawrence Kass:** writing – original draft, writing – review and editing. **Kay Leaming‐Van Zandt:** writing – original draft, writing – review and editing. **Joan Noelker:** writing – original draft, writing – review and editing. **Erin McDonough:** writing – original draft, writing – review and editing.

## Funding

The authors have nothing to report.

## Disclosure

Presentations: Concept developed from original presentation at the SAEM Annual Meeting in May 2025 titled “Developing a Clinical Skills Training Framework for Emergency Medicine Faculty.”

## Conflicts of Interest

The authors declare no conflicts of interest.

## Data Availability

Data sharing not applicable to this article as no datasets were generated or analysed during the current study.
